# Facilitating Lithium-Ion Diffusion in Layered Cathode Materials by Introducing Li^+^/Ni^2+^ Antisite Defects for High-Rate Li-Ion Batteries

**DOI:** 10.34133/2019/2198906

**Published:** 2019-09-15

**Authors:** Zhongfeng Tang, Sen Wang, Jiaying Liao, Shuo Wang, Xiaodong He, Bicai Pan, Haiyan He, Chunhua Chen

**Affiliations:** ^1^CAS Key Laboratory of Materials for Energy Conversions, Department of Materials Science and Engineering & Collaborative Innovation Center of Suzhou Nano Science and Technology, University of Science and Technology of China, Anhui Hefei 230026, China; ^2^School of Physical Sciences, University of Science and Technology of China, Anhui Hefei 230026, China

## Abstract

Li^+^/Ni^2+^ antisite defects mainly resulting from their similar ionic radii in the layered nickel-rich cathode materials belong to one of cation disordering scenarios. They are commonly considered harmful to the electrochemical properties, so a minimum degree of cation disordering is usually desired. However, this study indicates that LiNi_0.8_Co_0.15_Al_0.05_O_2_ as the key material for Tesla batteries possesses the highest rate capability when there is a minor degree (2.3%) of Li^+^/Ni^2+^ antisite defects existing in its layered structure. By combining a theoretical calculation, the improvement mechanism is attributed to two effects to decrease the activation barrier for lithium migration: (1) the anchoring of a low fraction of high-valence Ni^2+^ ions in the Li slab pushes uphill the nearest Li^+^ ions and (2) the same fraction of low-valence Li^+^ ions in the Ni slab weakens the repulsive interaction to the Li^+^ ions at the saddle point.

## 1. Introduction

Lithium-ion batteries are experiencing the large applications in mobile electronic devices and electric vehicles worldwide. High-energy density and high-power density are the two most important factors in a commercial lithium-ion battery. For the cathode materials, compared to the widely used LiFePO_4_, LiMn_2_O_4_, and LiNi_1/3_Co_1/3_Mn_1/3_O_2_ [1–4], the layered nickel-rich materials with higher capacity (170-200 mAh g^−1^) and appropriate working voltage (~3.75 V), such as LiNi_0.6_Co_0.2_Mn_0.2_O_2_, LiNi_0.8_Co_0.1_Mn_0.1_O_2_ (NCM811), and LiNi_0.8_Co_0.15_Al_0.05_O_2_ (NCA) [5–8], have attracted more and more attention.

One of the challenging problems of layered nickel-rich cathode materials is the Li^+^/Ni^2+^ antisite defects which result from their similar ionic radii (Li^+^: 0.072 nm, Ni^2+^: 0.069 nm) [9, 10]. It is very difficult to synthesize a pure LiNiO_2_ phase because a high-temperature treatment leads to the phase transition from LiNiO_2_ to Li_1‐*x*_Ni_1+*x*_O_2_ which has a partially disordered cation distribution at the lithium layer, while a low-temperature treatment cannot bring sufficient crystallinity [11]. Although the cosubstitution of Co and Mn or Al for Ni can greatly inhibit the LiNiO_2_ phase change, there is still some cation disordering in Ni-rich layered materials because Ni^2+^ ions cannot be completely oxidized to Ni^3+^ even under oxygen-rich atmospheres [12, 13]. If there are a lot of Ni^2+^ ions in the lithium layer (i.e., 3b sites), the diffusion of lithium ions is inevitably hindered in the two-dimensional channels, which is why the researchers desire to decrease the cation disordering in layered materials [14–16].

Nevertheless, the Li^+^/Ni^2+^ antisite defects are not always detrimental to layered materials. Zheng et al. found that these defects benefit the thermal stability for Ni-rich NCM materials, because the Ni in the Li layer would form 180° Ni−O−Ni super exchange chains [17]. Moreover, it can partially relieve the magnetic frustration by forming a stable antiferromagnetic state in hexagonal sublattice with nonmagnetic ions located in centers of the hexagons [18]. Lee et al. have proved that a little amount of Ni^2+^ ions occupying Li^+^ (3b) sites is not a major obstacle to the diffusion of Li^+^ ions in the lithium layer [19]. In some cases, the substitution of inactive ions for Li^+^ or Na^+^ in the layered cathode materials can even improve the electrochemical properties due to the inhibition of phase transition and the enlargement of lattice parameters [20–22]. On the other hand, for the ions at 3a sites, Kang et al. indicated that [23] lower valence transition metal ions (Cu^2+^ or Ni^2+^ vs. Co^3+^ or Mn^4+^) can result in a weaker electrostatic interaction between Li^+^ in the activated state and the transition metal ions at 3a sites and thus substantially decrease the activation barrier for the migration of Li^+^ ions to the nearest vacancies. Hence, it is reasonable to speculate that the Li^+^ ions at 3a sites can further decrease the activation barrier for the diffusion of Li^+^ (3b) ions. Furthermore, a Ni^2+^ ion at 3b site in the lithium layer may also have a bigger electrostatic interaction (repulsion) with the nearest six Li^+^ ions, which can increase the energy of the activated lithium ions and thus decrease the activation barrier for lithium-ion migration.

Herein, taking NCA as an example of the Ni-rich cathode materials, we investigated the relationships between the rate capability and the Li^+^/Ni^2+^ antisite defects by combining experimental and theoretical calculations. Our experimental results indicate that the NCA sample with 2.3% Li^+^/Ni^2+^ exchange has the highest Li^+^ diffusion coefficient and exhibits the best rate capability, and our calculation results reveal that the activation barrier for the diffusion of Li^+^ ions significantly decreases when there is a minor degree of Li^+^/Ni^2+^ antisite defect in the Ni-rich layered oxides.

## 2. Materials and Methods

### 2.1. Synthesis of NCA Materials

The NCA samples were synthesized by a thermal polymerization method as described in our previous studies [24]. Typically, lithium nitrate (LiNO_3_), nickel nitrate (Ni(NO_3_)_2_·6H_2_O), cobalt nitrate (Co(NO_3_)_2_·6H_2_O), and aluminum nitrate (Al(NO_3_)_3_·9H_2_O) were dissolved in deionized water to prepare the precursor solutions. The molar ratio of Li, Ni, Co, and Al was 1.00 : 0.8 : 0.15 : 0.05, and the metal (Li+Ni+Co+Al) concentration was 1.0 mol L^−1^. Then, acrylic acid (AA, CH_2_=CHCOOH) was added into the mixture as a complexing agent, and the volume ratio of AA and H_2_O is 1 : 2. The mixed solution was kept at 160°C for 6 h to form xerogel, followed by thoroughly grinding and sintering at 500°C in air to remove the organics. The obtained precursor powders were again sintered at a tube furnace with flowing oxygen atmosphere for 12 h, and the sintering temperatures were set as 720°C, 735°C, 750°C, 765°C, and 780°C, respectively. For convenience, the prepared samples were denoted as NCA-720, NCA-735, NCA-750, NCA-765, and NCA-780, respectively.

### 2.2. Structure and Morphology Characterizations

Morphological studies were performed using scanning electron microscopy (SEM, JSM-6390LA, JEOL). The crystal structures of the prepared samples were measured by a theta/theta rotating anode X-ray diffractometer (XRD, Rigaku TTR-lll). The X-ray Rietveld refinement was performed by using a GSAS/EXPGUI package [25].

### 2.3. Electrochemical Analysis

Li-half 2032 coin cells were used to test the electrochemical properties. NCA powders (84 wt%), carbon black (8 wt%), and polyvinylidene fluoride (8 wt%) that dispersed in N-methyl-2-pyrrolidone were thoroughly mixed to make slurries. Then, the slurry was coated on an aluminum foil and dried at 80°C in an oven overnight. The laminates were punched into round disks with a diameter of 12 mm. A typical electrode disk contained 4 mg NCA. The coin cells were assembled in an Ar-filled glove box (MBraun Labmaster 130) with an electrolyte of 1 M LiPF_6_ in ethylene carbonate (EC) and diethyl carbonate (DEC) (EC : DEC = 1 : 1 *V*/*V*) and a separator of Celgard 2400 porous membrane.

The cells were charged/discharged on a Neware BTS 610 multichannel battery cycler at a constant current (0.1C-10C, 1C = 180 mAg^−1^) in the voltage range of 2.75-4.3 V for five cycles, respectively, to investigate the rate performance. In order to calculate the lithium-ion diffusion coefficient, the cyclic voltammograms (CV) were conducted on a CHI640 Electrochemical Workstation with a scan rate of 0.1, 0.3, 0.5, 0.8, and 1.0 mV s^−1^, respectively, between 2.5 and 4.5 V. And the impedance measurement was also conducted, at a fully discharged state, using electrochemical impedance spectroscopy (EIS) in the frequency range of 0.01 Hz-100 kHz. The amplitude of the alternating current (AC) signal was 5 mV.

### 2.4. Computational Method

**Figure 1:**
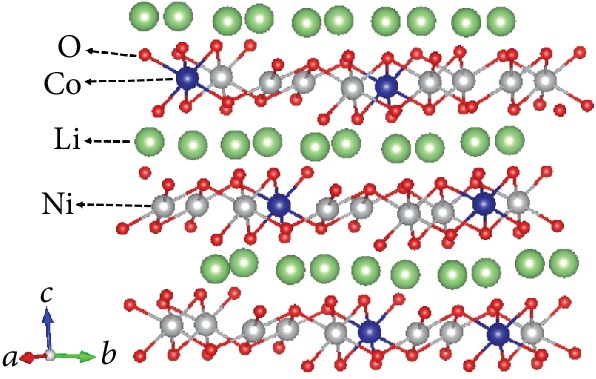
The structure of a 120-atom supercell consisting of 2 × 5 × 1 LiNi_0.8_Co_0.2_O_2_ primitive unit cells.

All calculations were performed by using a Vienna ab initio simulation package (VASP 5.3.3) based on the local density approximation [26]. The interaction between ions and valence electrons was described using projector augmented wave (PAW) potentials [27], and the on-site electron-electron interactions were taken into account by performing GGA+U level calculations with *U*‐*J* = 4.91 eV for Co-3d electrons and *U*‐*J* = 6 eV for Ni-3d electrons taken from the literatures [28–33]. A 120-atom supercell consisting of 2 × 5 × 1 primitive unit cells was employed, as shown in Figure 1, and all calculations were performed with a plane wave cutoff of 500 eV. In addition, we found a *k*-point sampling of 5 × 2 × 2 within the Monkhorst-Pack special *k*-point scheme in the Brillouin zone sufficiently dense for the bulk unit cell [34]. The energy relaxation iterates until the forces acting on all the atoms were less than 10^−2^ eV/Å. The climbing image nudged elastic band (CI-NEB) method was used to determine the minimum energy paths for diffusion of Li atoms in LiNi_0.8_Co_0.2_O_2_ [35–37]. With these settings, we obtained the optimal crystallographic parameters of LiNi_0.8_Co_0.2_O_2_, *a* = 2.79 *Å*, *b* = 2.95 *Å*, and *c* = 14.09 *Å*, which are in good agreement with the experimental values (*a* = 2.87 *Å*, *b* = 2.87 *Å*, and *c* = 14.13 *Å*) [38].

## 3. Results

### 3.1. Morphology and Structure Characterization

**Figure 2:**
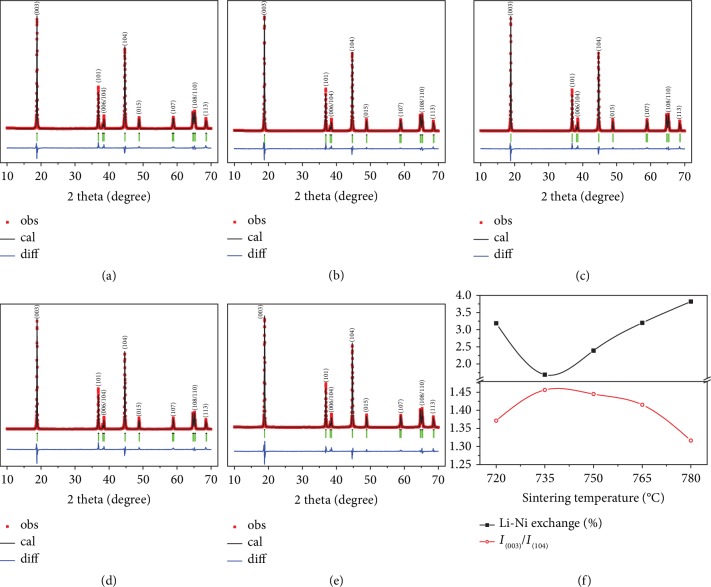
Rietveld refinement of XRD. (a–e) Rietveld analysis of NCA-720, NCA-735, NCA-750, NCA-765, and NCA-780, respectively. (f) The refinement results of the ratio of Li/Ni exchange and the peak intensity ratio of (003)/(104).

[Supplementary-material supplementary-material-1] shows the SEM images of the NCA samples sintered at different temperatures, which consist of irregular particles around 700 nm-1 *μ*m and without significant differences in the morphology and particle size. As indicated in Figures 2(a)–2(e), all their diffraction peaks can be well indexed in a hexagonal structure of *α*-NaFeO_2_ type without any impurity [39, 40]. In order to reveal the detailed crystal information, the XRD data have been refined by the Rietveld method using GSAS software. In the refinement, Li_1_/Ni_2_ are set at 3b sites (0, 0, 0.5), Li_2_/Ni_1_/Co_1_/Al_1_ at 3a sites (0, 0, 0), and O at 6c sites (0, 0, *z*) where *z* = 0.25 [41]. For the five samples, the average valences of transition metals (Ni and Co) are almost the same according to the semiquantitative analysis of X-ray photoelectron spectroscopy (XPS) (Figures [Supplementary-material supplementary-material-1] and [Supplementary-material supplementary-material-1]). For the cation disordering, only Li^+^ and Ni^2+^ occupations are considered to be exchanged because Co^3+^ (0.061 nm) and Al^3+^ (0.054 nm) in the lithium layer would cause a drastically increased system energy due to the big differences in ionic radii and valences with Li^+^. The refinement results are given in Table 1 and Figure 2(f), according to which two crystal diagrams of the TM layer and the Li layer (seen from *c*-axis direction) for NCA-750 are given in [Supplementary-material supplementary-material-1] to show the atom arrangements. It is quite clear that the ratio of Li^+^/Ni^2+^ exchange is highly related to the intensity ratio of *I*_(003)_/*I*_(104)_ that has been used to qualitatively analyze the degree of cation mixing in layered materials. It is widely believed that the lower the degree of cation disordering, the better the electrochemical properties [12, 14–16, 42]. NCA-720 has a relatively high degree of Li/Ni mixing because the temperature is not high enough to remove all of the lattice defects. The rest of the samples show an approximate linear relationship between the ratio of Li/Ni exchange and sintering temperature, which may be attributed to the phase transition of LiNiO_2_ with increasing sintering temperature. It seems that NCA-735 which has the least Li/Ni exchange in the layered structure should have exhibited the best electrochemical properties. However, our electrochemical measurements give us some anomalous but interesting results as shown in the following part.

### 3.2. Electrochemistry

**Figure 3:**
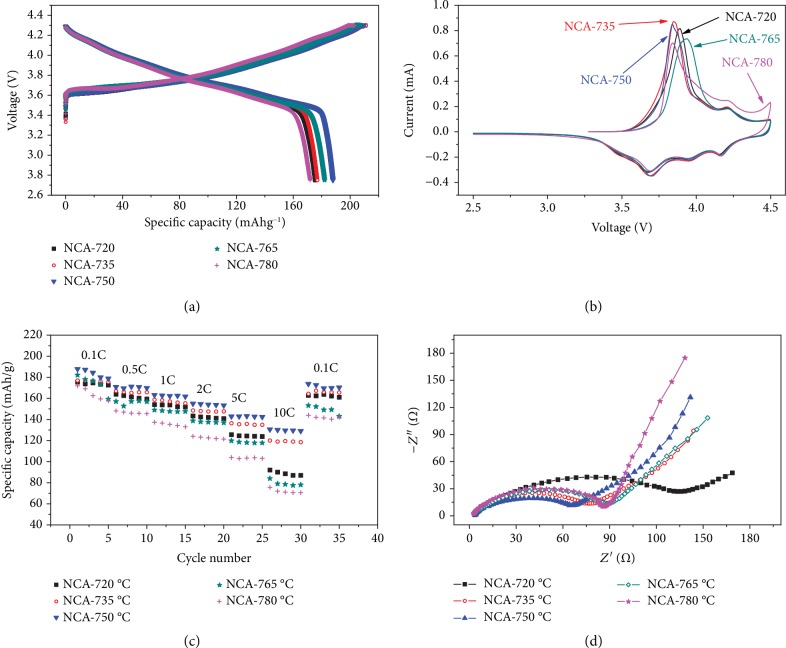
Electrochemical measurements and characterization: (a) the initial charge-discharge profiles; (b) the initial CV curves; (c) rate capabilities; and (d) Nyquist plots of Li/NCA half cells for the five samples.

The electrochemical properties of NCA electrode materials are shown in Figure 3. In Figure 3(a), the charge/discharge profiles are obtained at 0.1C in the range of 2.75-4.3 V. Obviously, the discharge capacity depends highly on the sintering temperature. NCA-750 achieves the highest initial discharge capacity of 187.9 mAh g^−1^ as well as the highest initial Coulombic efficiency of 89.5%. The CV curves with a scan rate of 0.1 mV s^−1^ in Figure 3(b) show the redox behaviors of the transition metals as well as the phase transitions during the charging/discharging process. All the samples show the similar oxidation of Ni^3+^ to Ni^4+^ at around 3.8 V but with slight difference in the oxidation peaks which may be caused by the larger polarization for NCA-720 and NCA-765. It should also be noted that NCA-780 has another oxidation peak at around 4.5 V which mainly is attributed to the phase transition from H1 to O1 [43]. As mentioned before, the LiNiO_2_-based materials decompose at high temperatures and form the nonstoichiometric Li_1‐*x*_NiO_2_ phase. And that is the main reason for its lowest capacity in the voltage range of 2.75-4.3 V. As shown in Figure 3(c), the rate performances differ significantly with different sintering temperatures. NCA-750 shows the best rate capability even with a capacity of 129.8 mAh g^−1^ at 10C rate while it is only 119.5 mAh g^−1^ for NCA-735. And the EIS also shows the results consistent with the rate performance (Figure 3(d)). These results and the diffusion coefficient of lithium ions (see below) indicate that an appropriate amount of Li^+^/Ni^2+^ antisite defects in the layered structure is beneficial to the fast diffusion of Li^+^ in the lithium layer.

**Figure 4:**
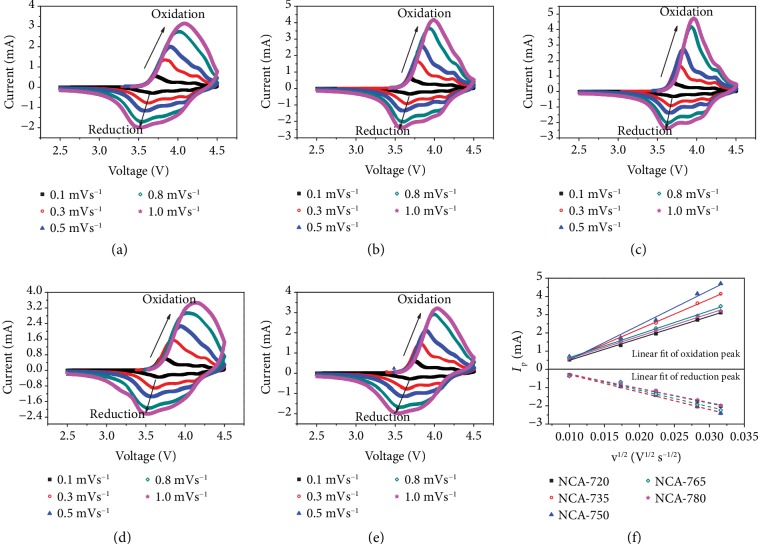
CV measurements. (a–e) CV curves of NCA-720, NCA-735, NCA-750, NCA-765, and NCA-780, respectively; (f) linear fit of the peak currents and the square root of scanning rates for the five samples.

The lithium-ion diffusion coefficient was calculated based on the CV curves with different scan rates (Figure 4) [44]. In a diffusion controlled process, the peak current is proportional to the square root of the scanning rates, which is in line with the Randles-Sevcik equation:

(1) $$\begin{array}{c} i_{\text{p}}=2.69*10^{5}*n^{3/2}C_{\text{Li}}{Av}^{1/2}{D_{\text{Li}}}^{1/2}, \end{array}$$

where *i*_p_ is the peak current, *n* is the number of the electrons transferred, *C*_Li_ is the concentration of Li^+^, *A* is the surface area of the electrode, *v* is the scan rate, and *D*_Li_ is the diffusion coefficient of lithium ions. As shown in Figures 4(a)–4(e), the deintercalation *O*‐*D*_Li_ and intercalation *R*‐*D*_Li_ are calculated from the oxidation peak and reduction peak, respectively, and the data are summarized in Table 2. The slope of the linear fit of each peak, as shown in Figure 4(f), is positively proportional to the diffusion coefficient of lithium ions. Clearly, the diffusion coefficient of the deintercalation process has a higher dependence on temperature, which is consistent with the rate performance, than that of the intercalation process.

### 3.3. Calculation Results

In order to understand the anomalous experimental result that NCA-750 with higher Li/Ni antisite defects show better electrochemical properties, especially the rate capability, than that of NCA-735 which contains the lowest antisite defects, we perform the calculations from the perspective of activation barrier which strongly affects the diffusion of lithium ions in electrode materials. However, due to the limited calculation abilities, we can only build a 120-atom supercell consisting of 2 × 5 × 1 LiNi_0.8_Co_0.2_O_2_ primitive unit cells as mentioned before but not a LiNi_0.8_Co_0.15_Al_0.05_O_2_ supercell in which the five percent aluminum ions would need a huge amount of computations.

**Figure 5:**
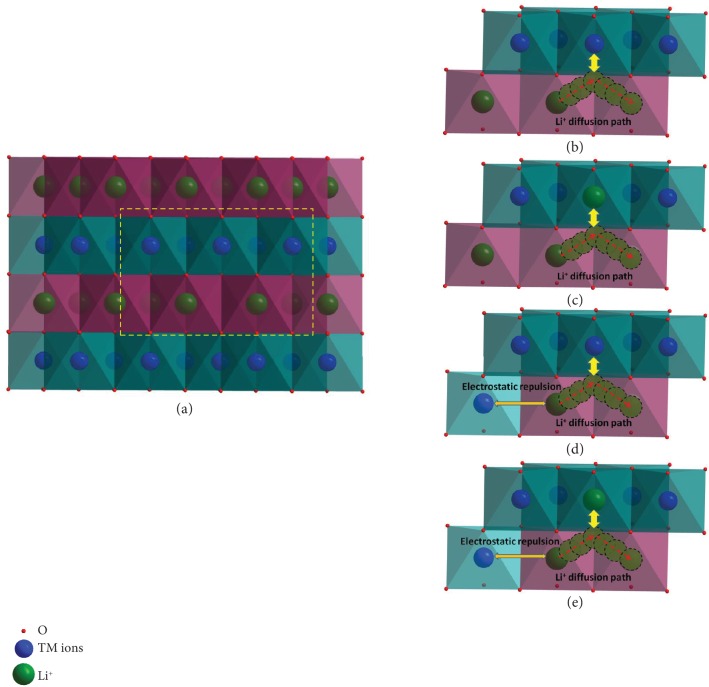
Structure and diffusion path of Li^+^ ions of the Ni-rich cathode materials. (a) Layered structure of the Ni-rich cathode materials; the diffusion of Li^+^ ions under different conditions: (b) without Li/Ni antisite defects, (c) Li ion occupies TM ion sites, (d) Ni^2+^ occupies Li^+^ sites; (e) simultaneously with two defects near the diffusion pathway.

In a typical layered structure as shown in Figure 5(a), the lithium ions and transition metal ions alternately occupy a layer of octahedron sites, which means that there is a two-dimensional transfer pathway for lithium ions. When a lithium ion migrates to the nearest lithium vacancy, it has to pass through the adjacent tetrahedron sites, in which the neighboring transition metal ion hinders the Li^+^ diffusion because of the strong electrostatic repulsion (Figure 5(b)) [23]. And that is the main reason for the activation barrier in layered electrode materials. If the high-valence TM ions are replaced by the Li^+^ ions, the activation barrier for Li^+^ diffusion decreases due to the weaker electrostatic repulsion (Figure 5(c)). On the other hand, when a high-valence TM ion occupies Li^+^ sites, i.e., Li/Ni exchange (Figures 5(d) and 5(e)), it should also have some effect on the activation barrier for the six nearest lithium ions' diffusion, which will be discussed in the following part in detail.

**Figure 6:**
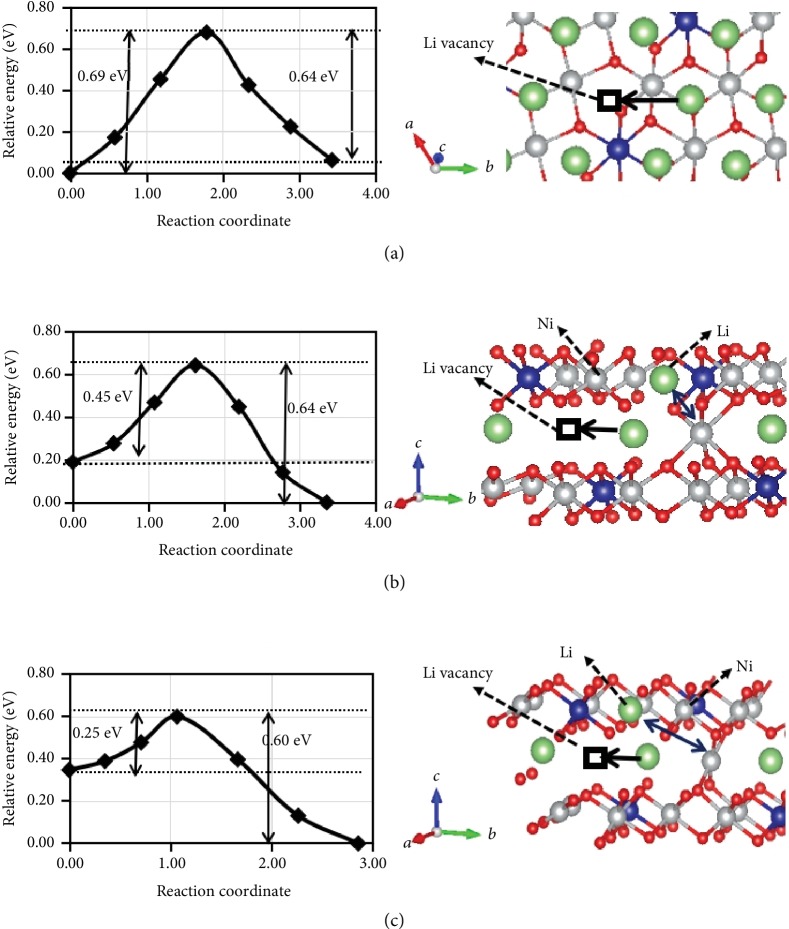
Calculation results of the activation barriers in lithium vacancy environments. (a) Energy profiles for Li diffusion and diffusion path in LiNi_0.8_Co_0.2_O_2_, (b) energy profiles for Li diffusion in LiNi_0.8_Co_0.2_O_2_ with 4.17% Li-Ni exchange and diffusion path under Ni ion, (c) energy profiles for Li diffusion in LiNi_0.8_Co_0.2_O_2_ with 4.17% Li-Ni exchange and diffusion path under Li ion. Squares are lithium vacancies, black arrows represent diffusion path, and the blue double-sided arrows represent Li-Ni exchange.

We then calculate the activation barriers in lithium vacancy environments, i.e., a lithium ion migrates to occupy its nearest lithium vacancy in Li slab, as shown in Figure 6. This is similar to the Li diffusion in LiCoO_2_ [45]. Firstly, we explore Li diffusion in LiNi_0.8_Co_0.2_O_2_ without Li-Ni exchange and find that the activation barriers are approximately 0.69 eV, as shown in Figure 6(a). Then, we consider the Li diffusion in LiNi_0.8_Co_0.2_O_2_ with 4.17% Li-Ni exchange, i.e., one Ni for one Li in the 120-atom supercell. The initial site of a Li ion is adjacent to a Ni ion in the Li slab, and the diffusion path is under the Ni ion in Ni(Co) slab, as shown in Figure 6(b). The calculated activation barriers decrease to be 0.45 eV, indicating that Li-Ni exchange favors the diffusion of Li. For this ratio of Li-Ni exchange, we also concern another case of Li diffusion path; that is, a diffusing Li ion is under an exchanged Li ion in the Ni(Co) slab, as shown in Figure 6(c). Surprisingly, the activation barrier decreases to 0.25 eV. Moreover, we further investigate diffusion of Li at the next neighboring Ni ion in the Li slab and find that the activation barriers are almost the same as those in LiNi_0.8_Co_0.2_O_2_ without Li-Ni exchange. This means that the Ni ions in the Li slab just influence the diffusion of Li ions within the scope of the neighboring region.

**Figure 7:**
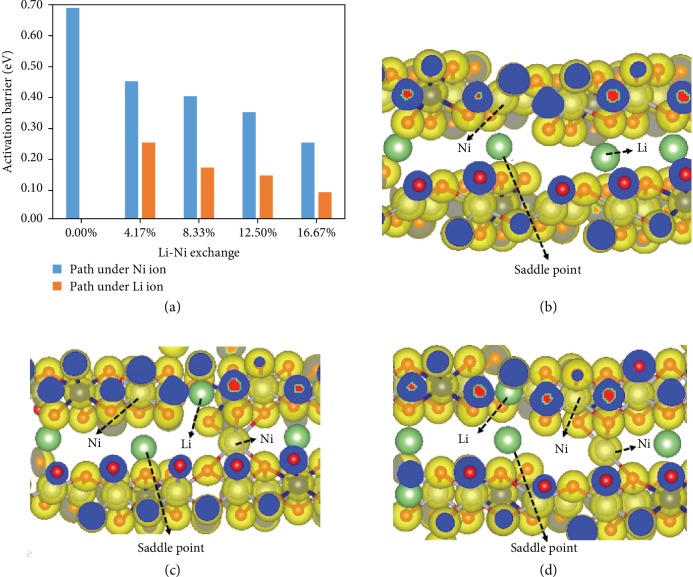
Calculation results of the activation barriers in Li-Ni exchange environments. (a) Calculated activation barrier for Li migration with different Li-Ni exchanges under Li or Ni ion, (b) charge distribution for Li diffusion in LiNi_0.8_Co_0.2_O_2_ without Li-Ni exchange, (c) charge distribution for Li diffusion in LiNi_0.8_Co_0.2_O_2_ with 4.17% Li-Ni exchange and diffusion path under Ni ion, and (d) charge distribution for Li diffusion in LiNi_0.8_Co_0.2_O_2_ with 4.17% Li-Ni exchange and diffusion path under Li ion.

Then, we focus on Li diffusion in LiNi_0.8_Co_0.2_O_2_ with different ratios of Li-Ni exchange. Our calculations reveal that the activation barrier further decreases with the increase of the ratio of Li-Ni exchange, as depicted in Figure 7(a). From Figure 7(a), we can see that for each concerned ratio of Li-Ni exchange, the activation barrier decreases by 0.2 eV at least when the diffusion path is under a Ni ion, as compared to that under a Li ion. Therefore, we can conclude that Li-Ni exchange promotes the diffusion possibilities of Li in LiNi_0.8_Co_0.2_O_2._

To understand this interesting phenomenon above, we investigate the electronic properties of the system. The charge distribution for the states at the saddle points of the diffusion paths for the typical cases is displayed in Figures 7(b)–7(d). We can see clearly from Figure 7(b) that the interactions between Li and O ions are weak while the interactions between Ni and O ions are strong in LiNi_0.8_Co_0.2_O_2_. When Li-Ni exchange occurs with a Ni ion appearing in the Li slab, stronger interactions between Ni ion in the Li slab and O ions drive Li ions near this Ni ion diffuse to its neighboring vacancies, and it reduces the activation barriers of Li diffusion around the Ni ion. This is why the Ni ions in Li slab can only alter the diffusion of Li ions within the scope of the neighboring region. For the same reason, when the Li diffusion path is under a Li ion, the above Li ion will reduce the interactions with surrounding O ions and then the distance between O ions and Li diffusion path, which results in the further decrease of activation barriers.

**Figure 8:**
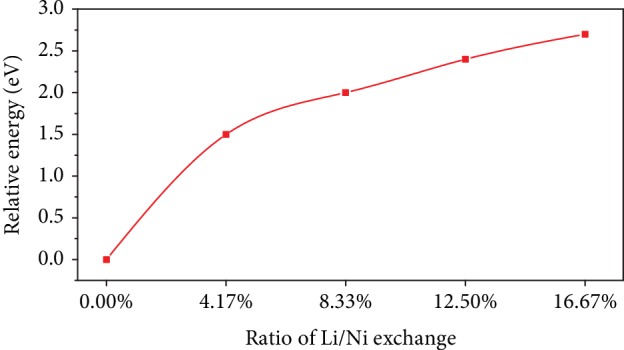
Change of total energy with different amounts of Li-Ni exchange in the supercell.

However, as shown in Figure 8, if there are too many Li-Ni antisite defects in Ni-rich layered materials, the total system energy drastically increases, which means that the layered structure becomes quite unstable with the increasing Li/Ni antisite defects. Therefore, it seems impossible to synthesize the layered structure materials with so many defects under the mentioned experimental conditions. On the other hand, the excessive inactive TM ions in Li slab would inevitably cause a longer diffusion path for lithium ions. Therefore, the highest rate of Li^+^ diffusion can be achieved when the benefit from the decrease of activation barriers and hindrance reach a balance, which corresponds to the experimental result of 2.39% for NCA-750 in our study.

## 4. Discussion

In this work, our experimental results show that, unlike the widely assumed, the layered Ni-rich material with an appropriate amount of Li/Ni antisite defects exhibits the best electrochemical properties. By building a 120-atom supercell consisting of 2 × 5 × 1 LiNi_0.8_Co_0.2_O_2_ primitive unit cells, we have investigated the relationships between the Li/Ni antisite defects and activation barrier for lithium ions' diffusion. When a Ni^2+^ ion occupies a Li^+^ site, the stronger interactions between Ni and O drive the nearest Li^+^ ion diffuse to the neighboring vacancies. In other words, high-valence TM ions can decrease the activation barrier for the diffusion of lithium ions, resulting in the improved rate capabilities. And the activation barrier can be further decreased when there is a synergy between Ni (3b sites) and Li (3a sites), i.e., Li/Ni exchange. However, a large amount of Li/Ni antisite defects cause the drastic increase of system energy and make the layered structure unstable. We expect that these interesting findings offer some opportunities to design the layered cathode materials for advanced lithium-ion batteries.

## Figures and Tables

**Table 1:** Summary of the Rietveld result of the five NCA samples based on the space group of R-3m.

Samples	*a* (Å)	*c* (Å)	*c*/*a*	*χ* ^2^	Rwp%	Rp%	*I*_(003)_/*I*_(104)_	Li/Ni exchange
NCA-720	2.8615 (4)	14.1738 (6)	4.9533	6.11	9.6	7.6	1.37131	3.19%
NCA-735	2.8614 (8)	14.1756 (4)	4.9541	7.629	10.3	7.6	1.45642	1.69%
NCA-750	2.8619 (3)	14.1776 (5)	4.9539	7.394	10.4	7.9	1.44492	2.39%
NCA-765	2.8628 (3)	14.1776 (3)	4.9539	9.188	11.4	8.5	1.41527	3.20%
NCA-780	2.8632 (1)	14.1781 (2)	4.9518	8.351	11	8.1	1.31637	3.82%

**Table 2:** The calculated diffusion coefficient of Li^+^ for deintercalation and intercalation processes.

Samples	*O*‐*D*_Li_ (cm^2^ s^−1^)	*R*‐*D*_Li_ (cm^2^ s^−1^)
NCA-720	5.79∗10^‐11^	2.49∗10^‐11^
NCA-735	1.08∗10^‐10^	3.63∗10^‐11^
NCA-750	1.45∗10^‐10^	3.66∗10^‐11^
NCA-765	6.54∗10^‐11^	3.26∗10^‐11^
NCA-780	5.51∗10^‐11^	2.16∗10^‐11^

## Supplementary Materials

Supplementary Materials Figure S1: SEM images of (a) NCA-720, (b) NCA-735, (c) NCA-750, (d) NCA-765, and (e) NCA-780, respectively. Figure S2: XPS spectra of (a) survey spectrum and Ni 2p for (b) NCA-720, (c) NCA-735, (d) NCA-750, (e) NCA-765, and (f) NCA-780, respectively. Figure S3: XPS spectra of Co 2p for (a) NCA-720, (b) NCA-735, (c) NCA-750, (d) NCA-765, and (e) NCA-780, respectively. Figure S4: crystal diagrams of the TM layer (a) and Li layer (b) for NCA-750.
