# Supplementary material for: Facilitating Lithium-Ion Diffusion in Layered Cathode Materials by Introducing Li+/Ni2+ Antisite Defects for High-Rate Li-Ion Batteries
Source: Research (Wash D C). 2019 Sep 15;2019:2198906. doi: 10.34133/2019/2198906 (PMC6946265; doi:10.34133/2019/2198906)
Supplement: Supplementary Materials — Figure S1: SEM images of (a) NCA-720, (b) NCA-735, (c) NCA-750, (d) NCA-765, and (e) NCA-780, respectively. Figure S2: XPS spectra of (a) survey spectrum and Ni 2p for (b) NCA-720, (c) NCA-735, (d) NCA-750, (e) NCA-765, and (f) NCA-780, respectively. Figure S3: XPS spectra of Co 2p for (a) NCA-720, (b) NCA-735, (c) NCA-750, (d) NCA-765, and (e) NCA-780, respectively. Figure S4: crystal diagrams of the TM layer (a) and Li layer (b) for NCA-750. [file 2198906.f1.docx]

**Facilitating Lithium Ions Diffusion in Layered Cathode Materials by Introducing Li^+^/Ni^2+^AntisiteDefects for High Rate Li-Ion Batteries**

Zhongfeng Tang^†,1^, Sen Wang^†,2^, Jiaying Liao^1^, Shuo Wang^1^, Xiaodong He^1^, Bicai Pan^2^, Haiyan He^*,2^, Chunhua Chen^^[[1]](#footnote-1)^*,1^

*^1^ CAS Key Laboratory of Materials for Energy Conversions, Department of Materials Science and Engineering & Collaborative Innovation Center of Suzhou Nano Science and Technology, University of Science and Technology of China, Anhui Hefei 230026, China*

*^2^ School of Physical Sciences, University of Science and Technology of China, Anhui Hefei 230026, China*


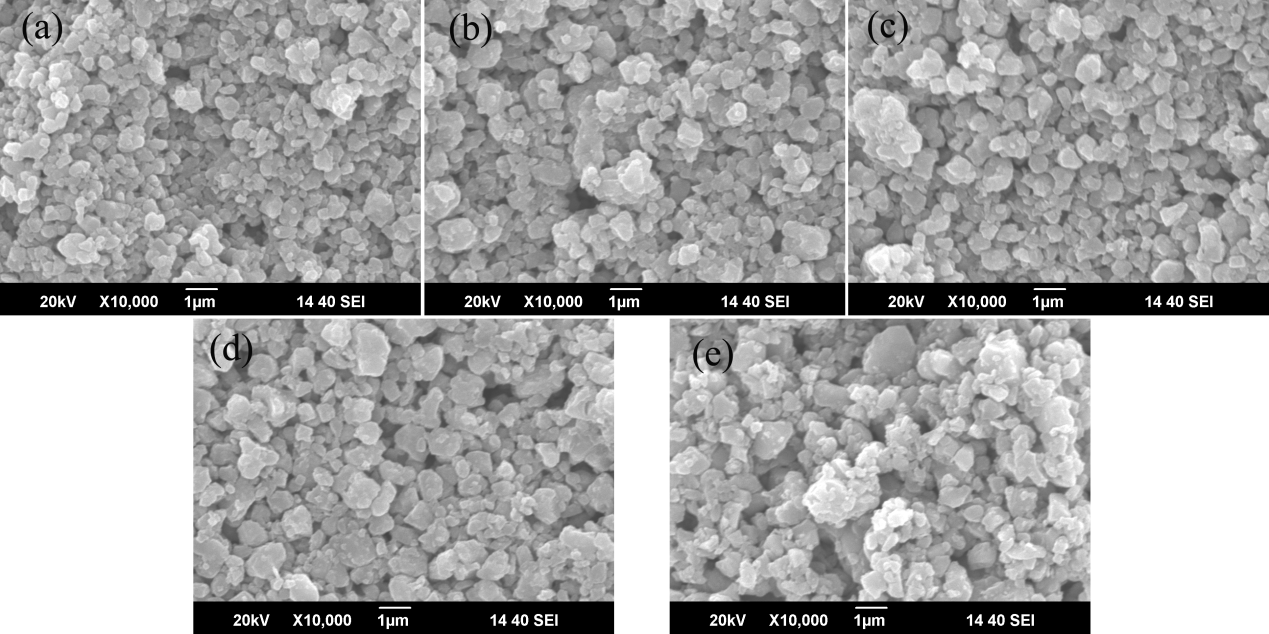


**Figure** S1. SEM images of (a) NCA-720, (b) NCA-735, (c) NCA-750, (d) NCA-765 and (e) NCA-780, respectively.


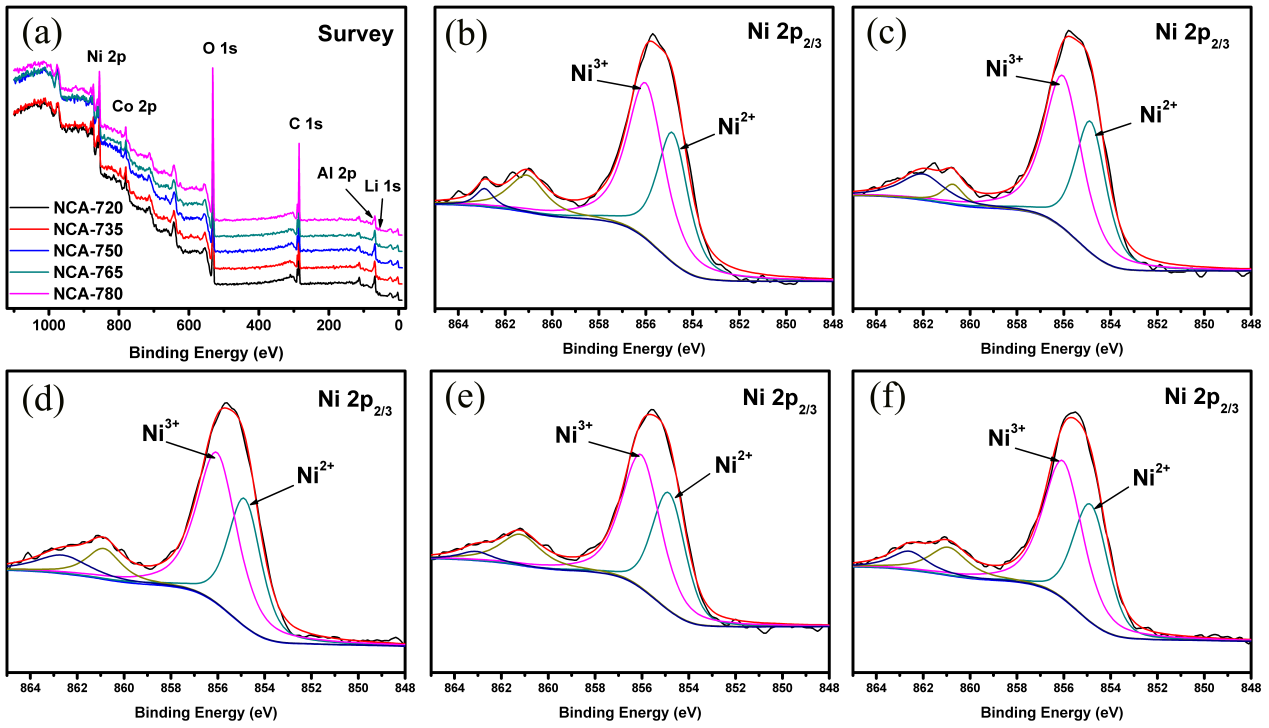


**Figure** S2. XPS spectra of (a) survey spectrum and Ni 2p for (b) NCA-720, (c) NCA-735, (d) NCA-750, (e) NCA-765 and (f) NCA-780, respectively.


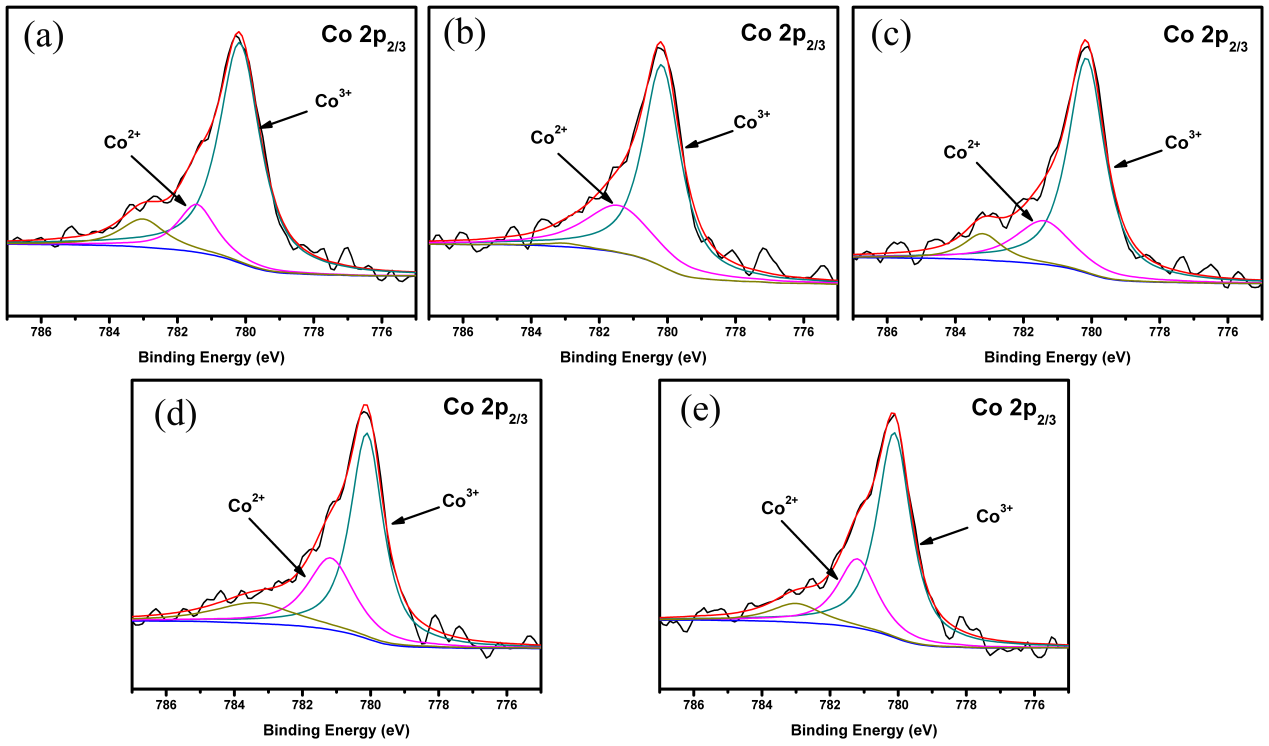


**Figure** S3. XPS spectra of Co 2p for (a) NCA-720, (b) NCA-735, (c) NCA-750, (d) NCA-765 and (e) NCA-780, respectively.


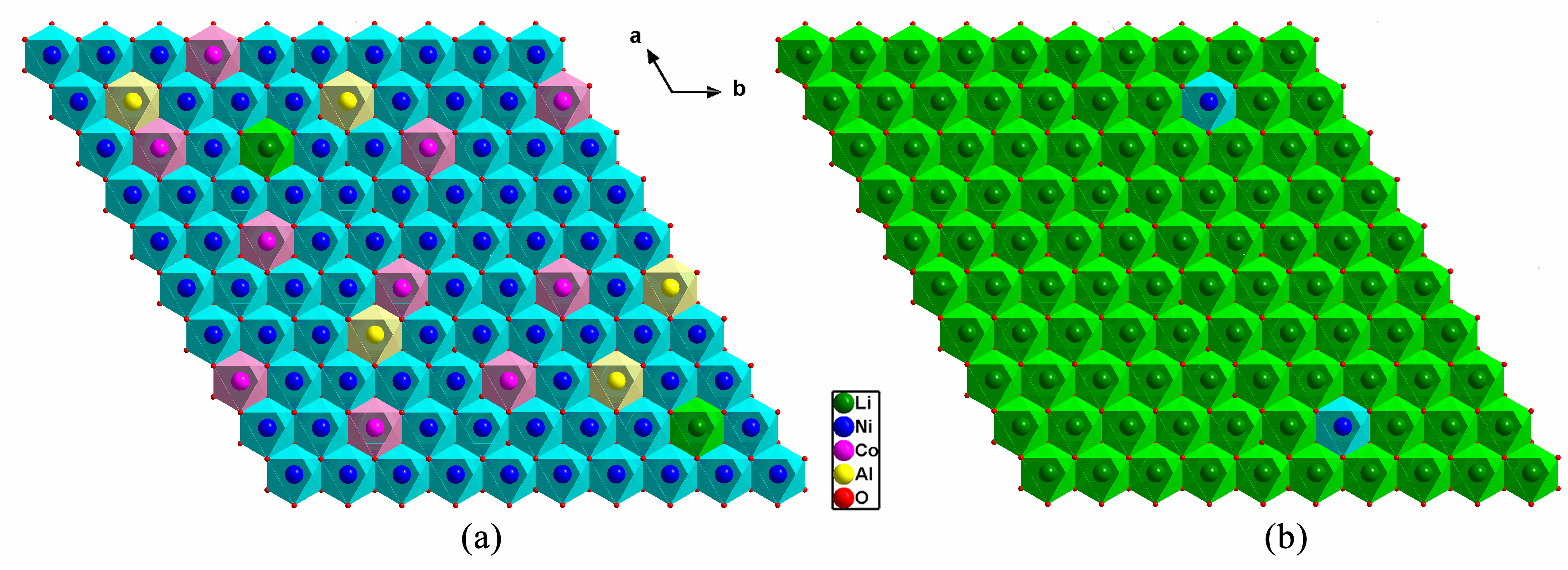


**Figure** S4. Crystal diagrams of the TM layer (a) and Li layer (b) for NCA-750.

1. * Corresponding author. Tel.: +86 551 63606971; fax: +86-551-63601952. *E-mail address:* cchchen@ustc.edu.cn (Chunhua Chen); [hyhe@ustc.edu.cn](mailto:hyhe@ustc.edu.cn) (Haiyan He).

   ^†^: Authors who contribute equally to this work. [↑](#footnote-ref-1)
